# Context-dependent AMPK activation distinctly regulates TAp73 stability and transcriptional activity

**DOI:** 10.1038/s41392-018-0020-y

**Published:** 2018-07-27

**Authors:** Dan Li, Iqbal Dulloo, Kanaga Sabapathy

**Affiliations:** 1 Division of Cellular & Molecular Research, Humphrey Oei Institute of Cancer Research, National Cancer Centre Singapore, Singapore, 169610 Singapore; 2 Cancer and Stem Cell Biology Program, Duke-NUS Graduate Medical School, Singapore, 169857 Singapore; 3 Department of Biochemistry, Yong Loo Lin School of Medicine, National University of Singapore, Singapore, 119228 Singapore; 4 Institute of Molecular & Cellular Biology, Singapore, 138673 Singapore; 5 Present Address: Sir William Dunn School of Pathology, University of Oxford, South Parks Road Oxford, Oxford, OX1 3RE UK

## Abstract

TAp73, the homologue of the tumour suppressor p53, has dual roles in tumourigenesis: both as a tumour suppressor and as a promoter of tumour growth. We have recently shown that hypoxia, a condition prevalent in tumours, results in the stabilisation of TAp73 through a mechanism involving HIF-1α-mediated repression of the E3 ligase *Siah1*. Elevated TAp73 in turn regulates the angiogenic transcriptional programme, exemplified by *vegf-A* activation, thereby promoting angiogenesis and tumour growth. To further understand hypoxia-mediated TAp73 regulation, we have focused on the Adenosine monophosphate (AMP)-dependent protein kinase (AMPK) signalling pathway induced by hypoxia. We show that hypoxia-mediated AMPK activation is required for efficient TAp73 stabilisation, through multiple means by using AMPK-deficient cells or inhibiting its activity and expression. Conversely, direct AMPK activation using its activator AICAR is also sufficient to induce TAp73 stabilisation but this is independent of putative AMPK phosphorylation sites on TAp73, HIF-1α activation, and transcriptional repression of *Siah1*. Furthermore, while *vegf-A* up-regulation upon hypoxia requires AMPK, direct activation of AMPK by AICAR does not activate *vegf-A*. Consistently, supernatant from cells exposed to hypoxia, but not AICAR, was able to induce tube formation in HUVECs. These data therefore highlight that the processes of TAp73 stabilisation and transcriptional activation of angiogenic target genes by AMPK activation can be decoupled. Collectively, these results suggest that the context of AMPK activation determines the effect on TAp73, and proposes a model in which hypoxia-induced TAp73 stabilisation occurs by parallel pathways converging to mediate its transactivation potential.

## Selective signalling stimulates tumour vascularisation

The stabilisation of an important signalling protein can fuel tumour growth and progression—but only under the right environmental conditions. Paradoxically, the TAp73 protein can both suppress tumorigenesis and stimulate formation of tumour-feeding blood vessels. The latter effect appears to be linked with exposure to oxygen-poor conditions within solid tumours, and researchers led by Kanaga Sabapathy at Singapore’s National Cancer Centre recently explored the mechanisms regulating TAp73 activity. Sabapathy’s team showed that the action of a protein called AMPK helps to prevent TAp73 from being degraded. However, activation of AMPK in isolation is insufficient to promote TAp73-mediated blood vessel growth. Instead, the researchers only observed this effect when AMPK was specifically activated due to oxygen deprivation, revealing the existence of multiple TAp73-regulating pathways that could explain this protein’s seemingly contradictory effects on cell growth.

## Introduction

p73, the homologue of the tumour suppressor p53, exists as two major forms: the full-length TAp73 form and the amino-terminally truncated, transactivation domain-deficient (DN)-p73 form.^1^ While DNp73 has been shown to be overexpressed in cancers and lead to resistance to cell death by virtue of its ability to inhibit both p53 and TAp73-dependent apoptosis^2–5^, TAp73’s role in carcinogenesis is more complex. While its apoptotic and tumour-suppressive roles have been firmly established,^2,6^ they do not concur with its overexpression in a larger number of human cancers where it is unmutated.^7,8^ This has led to the search for other non-tumour suppressive functions of TAp73, which has resulted in the revelation that TAp73 can indeed promote tumour cell growth in defined contexts. For instance, TAp73 can co-operate with proto-oncogenes such as c-Jun to transactivate AP-1 target genes like *cyclinD1* (refs. ^9,10^), or transactivate genes involved in the pentose phosphate pathway, to promote tumour cell proliferation.^11^ In addition, it was recently shown that TAp73 is capable of transactivating angiogenic target genes in response to hypoxia, thereby supporting tumour growth.^12,13^

Hypoxia, a condition that is prevalent in the core of solid tumours where oxygen supply is limited, results in enhanced angiogenesis, thereby allowing tumour cells to survive.^14,15^ Exposure of cells to hypoxia induces a myriad of signalling pathways which co-ordinately result in the ultimate survival of cancer cells.^16^ One among the many players that transmit the hypoxic signal is the hypoxia-inducible factor-1α (HIF-1α), which is a master regulator of angiogenic target genes including *vegf-A*.^17–20^ We have recently shown that hypoxia also leads to the stabilisation of the p73 proteins (both TAp73 and DNp73) (refs. ^12,13^). Mechanistically, hypoxia-mediated HIF-1α induction leads to the suppression of the E3 ligase *Siah1*, which otherwise leads to TAp73 degradation. Thus, under hypoxic conditions, TAp73 is relieved from Siah1-mediated degradation and in turn directly binds to angiogenic target gene promoters to transactivate them. In that study, we also noticed that multiple other E3 ligases are regulated by hypoxia and affect TAp73 levels,^12^ indicating that several parallel or overlapping pathways may be involved in regulating TAp73 stability upon hypoxia.

AMPK, a known regulator of cellular energy homeostasis, is induced by hypoxia.^21^ While activation of AMPK has been demonstrated to result in reduction of tumour cell growth,^22^ it has also been suggested to fuel the growth of cancers, classifying it as a contextual oncogene.^23^ In this context, AMPK has been shown to be a positive regulator of p53 stability,^24^ and a recent report has suggested that TAp73 can also be stabilised by AMPK, through direct binding and phosphorylation on the Serine residue 426 (ref. ^25^). Another report had suggested that treatment of neural cells with metformin, an AMPK activator, resulted in their proliferation in a TAp73-dependent manner.^26^ These reports together raise the possibility that AMPK activity may regulate p73 to support tumour growth in appropriate cellular settings. However, opposing views have also been put forth. An earlier study had suggested that AMPK can specifically bind to TAp73 alpha isoform, and lead to an inhibition of its transactivation ability.^27^ Hence, the role of AMPK in TAp73 stability and activity is at present unclear.

We have explored here the role of AMPK in the context of hypoxia-mediated TAp73 function. Our data suggest that AMPK is required for hypoxia-mediated TAp73 stabilisation and activity. However, its direct activation (out of the hypoxia context) – though sufficient to induce TAp73 stabilisation, is insufficient to activate TAp73’s transactivation potential on angiogenic genes such as *vegf-A*, indicating that the requirements for TAp73 stabilisation and activation can be decoupled. Moreover, TAp73 stabilisation upon direct AMPK activation is not dependent on HIF-1α, and does not affect the expression of *Siah1*, suggesting that multiple pathways work in concert to stabilise TAp73.

## Materials and methods

### Cell culture

p53-null human lung cancer cell lines H1299, and wild-type (WT) mouse embryonic fibroblasts (MEFs) or those lacking p73 (p73^−/−^); or AMPKα1 and α2 (AMPK double knockout, AMPK-DKO) or Itch (Itch^−/−^) were used in this study. Cells were grown in DMEM supplemented with 10% bovine foetal serum (FBS; Hyclone), 1% penicillin–streptomycin solution, 2 mM l-glutamine (Invitrogen, Carlsbad, CA, USA), 100 μM non-essential amino acids (Invitrogen) and 0.1 mM sodium pyruvate (Invitrogen), as described.^9^ Cells were treated with Compound C (Calbiochem), 5-aminoimidazole-4-carboxamide ribonucleotide (AICAR), dimethyloxalylglycine (DMOG) (Santa Cruz Biotechnology) at the indicated concentration or exposed to 1% oxygen to induce hypoxia as described.^12^ Human umbilical vein endothelial cells (HUVEC) were grown in M199 supplemented with 20% FBS, 1 μg/ml hydrocortisone, 20 μg/ml heparin sulphate, 250 ng/ml insulin. HCT116 cells, where the C-terminus of p73β isoform tagged with 3 × FLAG/His-tag was knocked into the p73 locus, were used as parental cells (TAp73β^+/+^) to generate TAp73^−/−^ cells by crispr-Cas9 system (unpublished data).

### siRNA, plasmids and transfections

Target sequence of siRNAs used: human HIF-1α: 5′-CTAACTGGACACAGTGTGT-3′. AMPKα1: 5′-GGUUGGCAAACAUGAAUUGTT-3′, AMPKα/2: 5′-GGUUUCUUAAAAACAGCUGTT-3′, and siRNA control (NegControl): 5′-UUCUCCGAACGUGUCACGUdTdT-3′, which were transfected using Transmessenger (Qiagen) following the manufacturer’s protocol. 48 h after siRNA transfection, the relevant plasmids were transfected using Lipofectamine Plus reagent, (Invitrogen) according to the manufacturer’s protocol. Expression vectors (all in pcDNA3) expressing full length p53, TAp73β, HIF-1α or dominant negative (DN) HIF-1α or the deletion mutants of TAp73β (1_425-TAp73, 1_399-TAp73), have been described previously.^12,28^ Wild type AMPK (WT-AMPK) and DN-AMPK plasmids have been described.^29^ TAp73β-S426A point mutation was generated by site-directed mutagenesis using the following primers: Forward: 5′-GGCATGAACAAGCTGCCCGCCGTCAACCAGCTGGTGGGC-3′; reverse: 5′-GCCCACCAGCTGGTTGACGGCGGGCAGCTTGTTCATGCC-3′. Luciferase reporter plasmids used include the following: *p21*-luc, *mdm2*-luc, *vegf-A*-luc, described previously.^12^

### RNA extraction and real-time RT-PCR

Total RNA was prepared from cells using TRIzol reagent (Invitrogen) according to the manufacturer’s instructions. 1.5–2 μg of total RNA was reverse transcribed into cDNA using SuperscriptII (Invitrogen). The sequences of real-time PCR primers and semi-quantitative reverse transcription-PCR analysis are the same as described.^12^

### Luciferase assays

H1299 cells were seeded in 6-well plates and transiently transfected with appropriate plasmids (0.5 μg) and β-galactosidase gene (50 ng) for normalisation. Cells were washed and lysed in luciferase lysis buffer. 24 h post-transfection, the luciferase assays were performed as described.^30^

### Immunoblot analysis

Cell lysates were prepared in lysis buffer containing 0.5% Nonidet P-40 as described.^12^ The total protein were quantified and then boiled in 4 × SDS sample buffer followed by separation on SDS polyacrylamide gels. Immunoblotting was performed with the antibodies, as follows: anti-Flag, anti-β-actin, anti-mouse secondary and anti-rabbit secondary antibodies (Sigma); anti-Myc, anti-phospho-AMPK and anti-AMPK (Cell signaling Technology); anti-Ubiquitin, anti-α-Tubulin and anti-EGFP (Santa Cruz Inc.); and anti-HIF-1α, anti-Itch (BD Transduction Laboratories). Ubiquitin-immunoprecipitation assay was performed as described.^12^ Size of bands are indicated in the blots.

### Tube formation assays

H1299 cells were seeded in 6-well plates and transiently transfected with appropriate plasmids pcDNA3.0 and TAp73β (0.5 μg). After 24 h post-transfection, the cells were treated with (DMSO), AICAR (1 μM), or exposed to hypoxia. 24 h thereafter, the conditioned medium was collected, and used to seed 5 × 10^4^/well HUVEC into wells of 96-well plate coated with matrigel. Images of the capillary network were taken after 6 h and the numbers of the loops were counted.^31,32^

### Statistical analysis

Data were analysed by two-way analysis of variance. The differences in mean values were considered significant at *p* values ≤0.01(**), and ≤0.05(*).

## Results

### Hypoxia-mediated TAp73 stabilisation requires AMPK activation

**Fig. 1:**
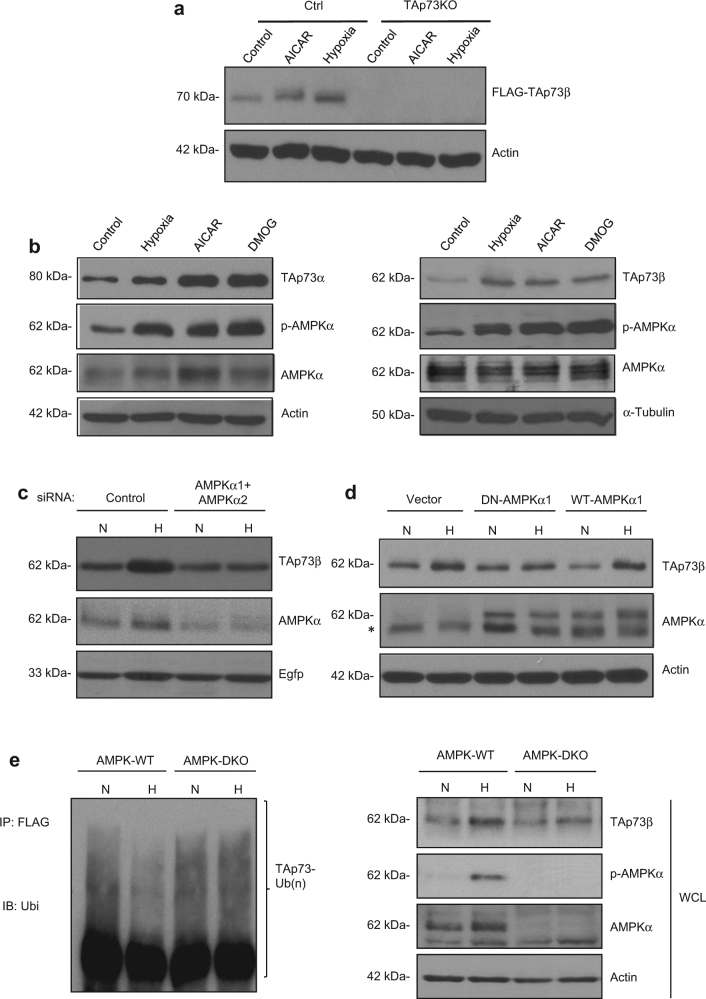
Hypoxia-mediated TAp73 stabilisation requires AMPK activation. **a** TAp73 proficient (Ctrl) or deficient (TAp73KO) HCT116 cells were treated with 1 mM AICAR, or incubated in 1% O_2_ (hypoxia) for 24 h, and the indicated protein levels were detected by immunoblotting (IB) with the corresponding antibodies. **b**–**d** p53-null H1299 cells were transfected with a Flag-TAp73α (left), or Flag-TAp73β (right) plasmid, and were treated with 1 mM AICAR, 1 mM DMOG, or incubated in 1% O_2_ (hypoxia) for 24 h, 24 h post-transfection, and the indicated protein levels were detected by IB with the corresponding antibodies (**b**). Similarly, control or AMPKα1/α2 siRNA (**c**) or the dominant negative (DN)-AMPK1 plasmid (**d**) were transfected together with the Flag-TAp73β plasmid and the expression of the indicated proteins were determined by IB after exposure to hypoxia for 24 h. WT-AMPKα1 plasmid was used as a control in (**d**). EGFP plasmid was transfected and serves as control for transfection as indicated. *: represent non-specific band. Parallel gels were run with similar amounts of lysates to determine the expression of the various proteins indicated, and representative loading controls are shown, unless specified throughout the manuscript. **e** Wild-type AMPK (AMPK-WT) or double knockout AMPK (AMPK-DKO) mouse embryonic fibroblasts (MEFs) were transfected with the Flag-TAp73β plasmid and analysed after hypoxia as described above by direct IB, or by ubiquitination assay after pull-down with anti-FLAG agarose beads followed by IB with anti-Ubi antibody (left panel). Ubiquitinated TAp73β is indicated as TAp73β-(Ub)n. Total TAp73 levels were detected by direct IB (right panel). For the ubiquitin assay, ubiquitin plasmid was transfected concurrently with the Flag-TAp73β plasmid. N normoxia, H hypoxia, WCL whole cell lystate. All immunoblotting experiments were repeated two to three times

We have previously shown that hypoxia results in the stabilisation of TAp73, leading to its pro-angiogenic activities.^12^ When analysing for pathways that can transmit the hypoxic signal to TAp73 stabilisation - besides HIF-1α-mediated *Siah1* suppression that reliefs TAp73 degradation,^12^ we observed that in the similar cellular system, the AMPK pathway was also activated by hypoxia, as reported earlier.^21^ Cells exposed to AICAR, an AMPK activator,^33^ led to an increase in the endogenous levels of TAp73β, to similar extents as that induced by hypoxia (1% oxygen) (Fig. 1a). To examine if this is a general phenomenon on the major TAp73 isoforms and involves the AMPK pathway, we treated cells transfected with either TAp73α or TAp73β with AICAR, hypoxia, or DMOG, a cell permeable prolyl-4-hydroxylase inhibitor that mimics hypoxia (Fig. 1b). All treatments led to increased phosphorylation of AMPKα, concomitant to an increase in the steady-state levels of the transfected TAp73α or TAp73β. We have therefore used TAp73β in all subsequent studies. To determine if AMPK activation was necessary and contributes to hypoxia-mediated TAp73 stabilisation, we undertook three approaches. Firstly, we silenced the expression of AMPKα1 and AMPKα2, the two catalytic α subunits of AMPK,^34^ and evaluated TAp73 levels. While TAp73β levels increased in the control cells, AMPK silencing led to a significant reduction of the increase of TAp73β upon hypoxia (Fig. 1c). Next, we utilised the DN-AMPKα1 plasmid, which has been shown to inhibit AMPK activity.^29^ Overexpression of DN-AMPKα1 also markedly abrogated the increase of TAp73β levels upon hypoxia, unlike WT-AMPKα1 (Fig. 1d). Finally, we used the AMPK-DKO MEFs, in which TAp73β stabilisation upon hypoxia was also compromised (Fig. 1e, right panel). Of note, TAp73β stabilisation upon hypoxia was not completely abrogated in all the above cases, but was reduced significantly, indicating that AMPK activation is one pathway among others contributing to TAp73 stabilisation upon hypoxia. Furthermore, we also determined if TAp73β ubiquitination is affected in the AMPK-DKO cells in ubiquitin assays. While immunoprecipitation of TAp73β followed by immunoblotting with the anti-ubiquitin antibody led to a decrease in ubiquitination of TAp73β upon hypoxia in WT cells, this phenomenon was reduced in the AMPK-DKO cells (Fig. 1e, left panel), supporting a role for AMPK in hypoxia-mediated TAp73 stabilisation. Collectively, these results demonstrate that the AMPK pathway activation is required and contributes to TAp73 stabilisation upon hypoxia.

### Direct AMPK activation is sufficient for TAp73 stabilisation, in a HIF-1α-independent manner

**Fig. 2:**
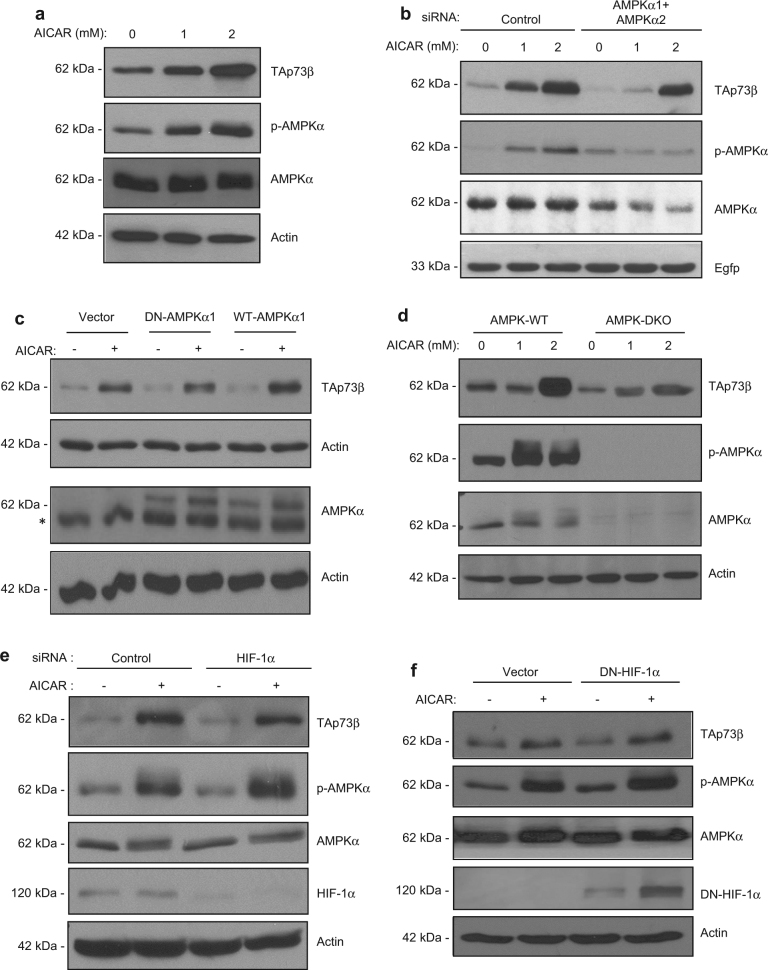
Direct AMPK activation by AICAR up-regulates TAp73 in a HIF-1α-independent manner. **a**–**f** H1299 cells were transfected with the Flag-TAp73β plasmid as described above and cells were treated with different concentrations of AICAR (**a**, **b**, **d**) or with 1 mM AICAR (**c**, **e**, **f**) for 24 h and lysates were used for IB. Control or AMPKα1/α2 siRNA (**b**); DN-AMPKα1 or WT-AMPKα1 plasmid (**c**); control or HIF-1α siRNA (**e**); and DN-HIF-1α plasmid (**f**) were transfected together with the Flag-TAp73β plasmid as indicated. AMPK-WT or AMPK-DKO MEFs were used in (**d**). *represent non-specific band. All immunoblotting experiments were repeated two to three times

We explored if there is an overlap between the AMPK-mediated pathway and the HIF-1α-dependent pathway described earlier,^17–21^ in TAp73 stabilisation. Treatment with the AMPK pathway activator AICAR led to a dose–dependent increase in AMPK phosphorylation, and a concomitant increase in TAp73β steady-state levels (Fig. 2a). Silencing AMPKα1 and AMPKα2 attenuated TAp73β stabilisation by AICAR, very significantly at low doses (Fig. 2b). At a higher AICAR concentration of 2 mM, TAp73β was still stabilised to a significant but however to a lesser extent than in the control cases. Expression of DN-AMPKα1 also led to an attenuation of the TAp73β stabilisation upon AICAR treatment, though this was not the case when WT-AMPKα1 was overexpressed (Fig. 2c). Furthermore, stabilisation of TAp73β was dramatically reduced in AMPK-DKO cells (Fig. 2d), confirming a critical role for AMPK in TAp73 stabilisation. We next examined if HIF-1α is essential for AICAR-mediated TAp73 stabilisation through AMPK. To this end, we first silenced the expression of HIF-1α concomitant to AICAR treatment (Fig. 2e). Silencing HIF-1α expression did not affect AMPK phosphorylation or stabilisation of TAp73β by AICAR. Similarly, overexpression of DN-HIF-1α also did not affect either of these events (Fig. 2f), suggesting that AICAR-induced AMPK signalling activation is sufficient for TAp73 stabilisation, and does not involve the HIF-1α pathway.

### AMPK activation does not contribute to Siah1 repression upon hypoxia

**Fig. 3:**
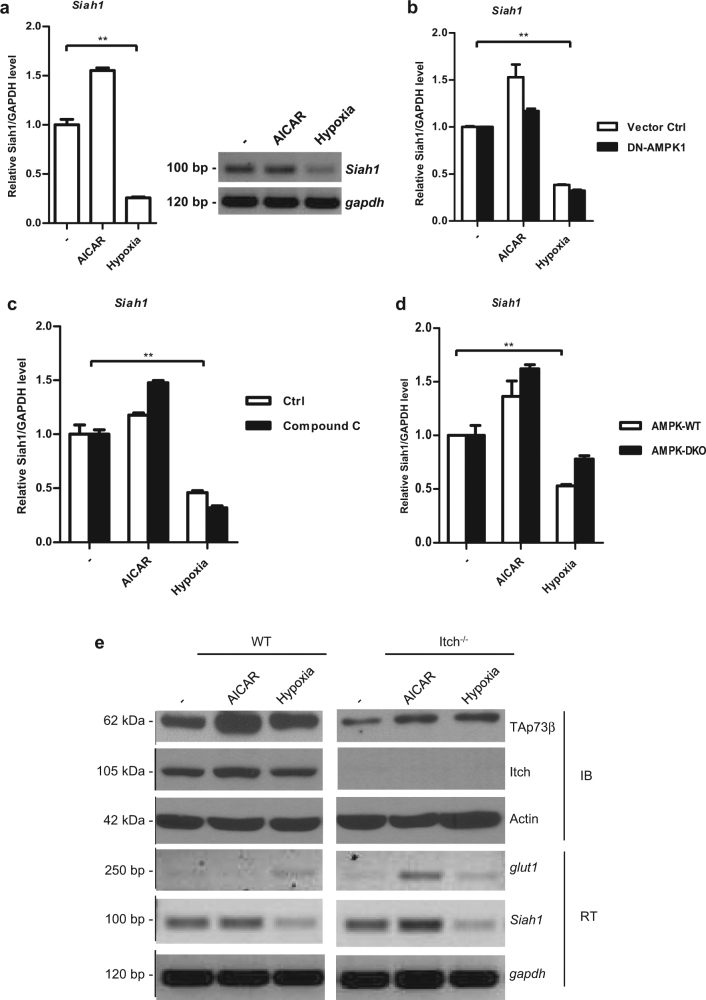
Hypoxia-mediated Siah1 repression is independent of AMPK. **a** Expression of *Siah1* in H1299 cells after exposure to hypoxia or treatment with 1 mM AICAR for 24 h was determined by semi-quantitative (right) or real-time quantitative (q) PCR (left). Relative levels of *siah1* to *gapdh* are shown, and the values of untreated samples are set to 1. Error bars indicate S.D. **p < 0.01. **b**–**d** H1299 cells were transfected with control vector or DN-AMPK1 plasmids (**b**), or were incubated in the presence of the AMPK inhibitor Compound C (**c**), and treated with AICAR or upon hypoxia as described above, and the levels of *Siah1* was determined by real-time qPCR analysis. Similarly, AMPK-WT or AMPK-DKO MEFs were used for analysis of *Siah1* (**d**). Error bars indicate S.D. **p < 0.01. **e** WT or Itch^−/−^ MEFs were transfected with the Flag-TAp73β plasmid and treated with AICAR or exposed to hypoxia for 24 h as described above, and the indicated protein levels were detected by IB. Parallel cultures were used to confirm *glut1* and *Siah1* levels by semi-quantitative RT-PCR (lower panels). All real-time PCR experiments were repeated two to three times and representative data from an experiment is shown. All immunoblotting experiments were repeated two to three times

Given that hypoxia has been shown to lead to TAp73 stabilisation through HIF-1α-mediated repression of the E3 ligase *Siah1* (ref. ^12^), and our current results which suggest that AMPK-mediated stabilisation of TAp73 by AICAR does not require HIF-1α, we examined if hypoxia-mediated AMPK activation pathway cross talks with the hypoxia-mediated HIF-1α-Siah1 axis. Firstly, we evaluated the effects on *Siah1* mRNA expression. While hypoxia repressed *Siah1* expression as previously reported,^12^ AICAR treatment did not, and even led to a marginal increase, as assessed by semi-quantitative PCR and quantitative real-time PCR (Fig. 3a). To further rule out the contribution of AMPK activation to hypoxia-mediated *Siah1* repression, we overexpressed DN-AMPKα1, which also did not have any appreciable effects on *Siah1* suppression upon hypoxia (Fig. 3b). Similar results were obtained when we used the AMPK-inhibitor, compound C^35^ (Fig. 3c). Finally, we also used AMPK-DKO cells, in which *Siah1* levels were still suppressed upon hypoxia, relatively similar to the WT counterparts (Fig. 3d). Finally, as it was recently suggested that AICAR treatment led to TAp73 stabilisation by inhibiting the E3 ligase Itch,^25^ we utilised Itch^−/−^ MEFs to evaluate the stability of TAp73 upon hypoxia and AICAR treatment. However, we observed that AICAR-mediated TAp73 increase was not significantly affected in Itch^−/−^ MEFs, and was similar to the increase upon hypoxia (Fig. 3e). Collectively, these results indicate that although AMPK is activated by hypoxia and contributes to TAp73 stabilisation, its activation does not impact hypoxia-mediated *Siah1* suppression, suggesting the existence of parallel pathways leading to TAp73 stabilisation upon hypoxia.

### S426 phosphorylation is not required for hypoxia and AMPK-mediated TAp73 stabilisation

**Fig. 4:**
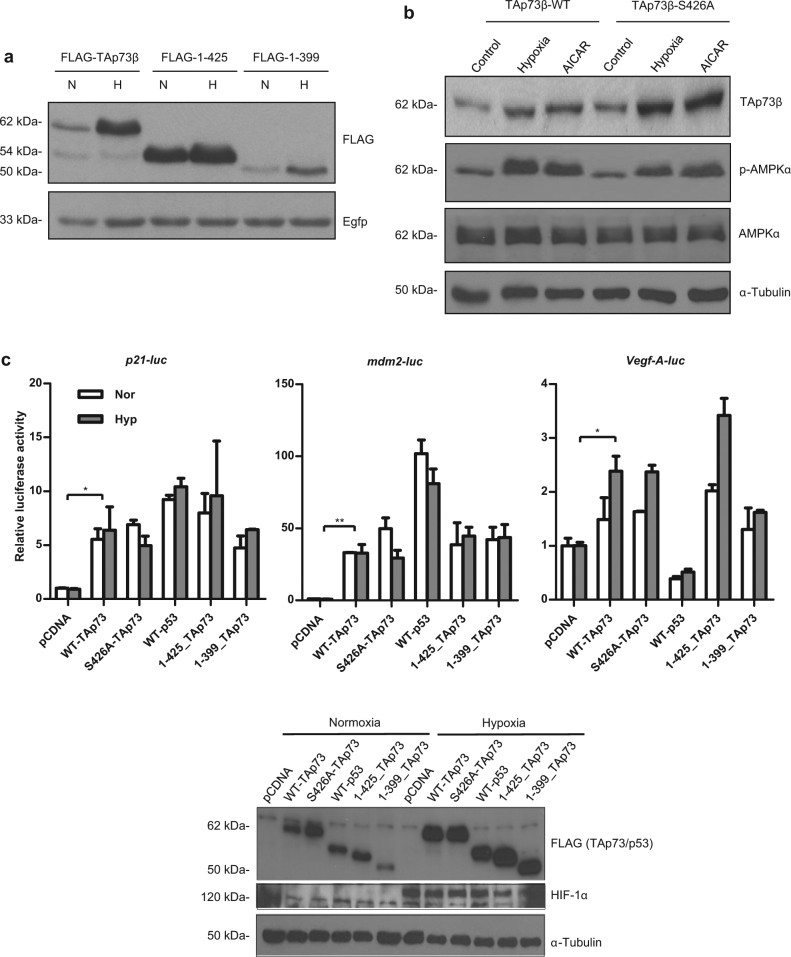
Phosphorylation of TAp73 at Serine 426 is not required for hypoxia or AICAR-mediated TAp73 stabilisation and activity. **a**, **b** H1299 cells were transfected with the indicated Flag-TAp73β truncation plasmids (**a**) or the Flag-TAp73β S426A mutant plasmid in which the Serine residue at amino acid 426 is substituted with an Alanine residue (**b**), and the levels of the indicated proteins were determined after hypoxia or AICAR treatment as described above. **c** Activation of *p21*, *Mdm2*, and *Vegf-A* promoter-luciferase activity was determined in H1299 cells transfected with different forms of TAp73 and wild type p53 (WT-p53), and exposed to hypoxia (upper panel). Lower panel shows the expression of the indicated proteins in lysates from one of the above reporter assays. Error bars indicate S.D. *p < 0.05; **p < 0.01. All immunoblotting experiments were repeated two to three times. All luciferase experiments were repeated two to three times, and representative data from an experiment is shown

A previous study has suggested that AMPK-mediated direct phosphorylation of TAp73 leads to its stabilisation.^25^ To further examine the requirements of AMPK-mediated TAp73 stabilisation, we utilised truncation constructs of TAp73 for further experiments. Using either the full length TAp73β, or the carboxyl-terminal truncated versions of amino acids (aa) 1–425 or 1–399 TAp73, we noticed that the steady-state levels of all the TAp73 forms were increased by hypoxia (Fig. 4a), despite varying basal levels. This suggested the earlier proposed phosphorylation site of S426 on TAp73 may be dispensable for AMPK or hypoxia-mediated TAp73 stability. To critically evaluate this, we generated a TAp73β construct in which the Serine 426 residue was mutated to a non-phosphorylable Alanine (S426A), and used it for further analysis. Treatment of p53 null H1299 cells transfected with either the WT or the S426A mutant constructs with hypoxia or AICAR revealed that there was no impediment on AMPK activation, as well as the increase in the steady-state levels of TAp73 due to the S426A alteration (Fig. 4b). We thus further examined if S426 is indeed not required for TAp73 functions upon hypoxia, by evaluating TAp73’s ability to regulate several target gene promoters driving the luciferase reporter gene. Expression of the various TAp73 carboxyl-terminus truncation mutants or the S426A mutant revealed that none of them were significantly compromised in their ability to regulate the classical p53/p73 responsive *p21* and *mdm2* promoters, and were on par with the WT TAp73β or p53 constructs under normoxic conditions (Fig. 4c). Moreover, hypoxia did not further enhance TAp73 or the various mutants’ ability to activate *p21* and *mdm2* promoters, despite the increase in their steady-state levels upon hypoxia. Similarly, all TAp73 mutants were also not compromised in their ability to activate the angiogenic *vegf-Α* promoter, and retained their ability to further enhance *vegf-Α* promoter activity upon hypoxia, as previously reported, highlighting specificity among target promoters that are regulated by TAp73 upon hypoxia. p53 was unable to activate *vegf-Α* as earlier noted.^12,36^ Moreover, the 1–399 TAp73 was unable to further activate the *vegf-Α* promoter upon hypoxia, suggesting the presence of other regulatory regions between the amino acids 399–425 of TAp73 that are necessary for this process. Nonetheless, the S426A mutant was as active as WT TAp73β in its ability to activate the targets genes, and in particular, the *vegf-Α* promoter upon hypoxia, indicating the hypoxic response does not require S426 phosphorylation for TAp73 stabilisation and activity.

### Direct AMPK activation is insufficient to transactivate vegf-A, but is required for efficient hypoxia-mediated vegf-A expression

**Fig. 5:**
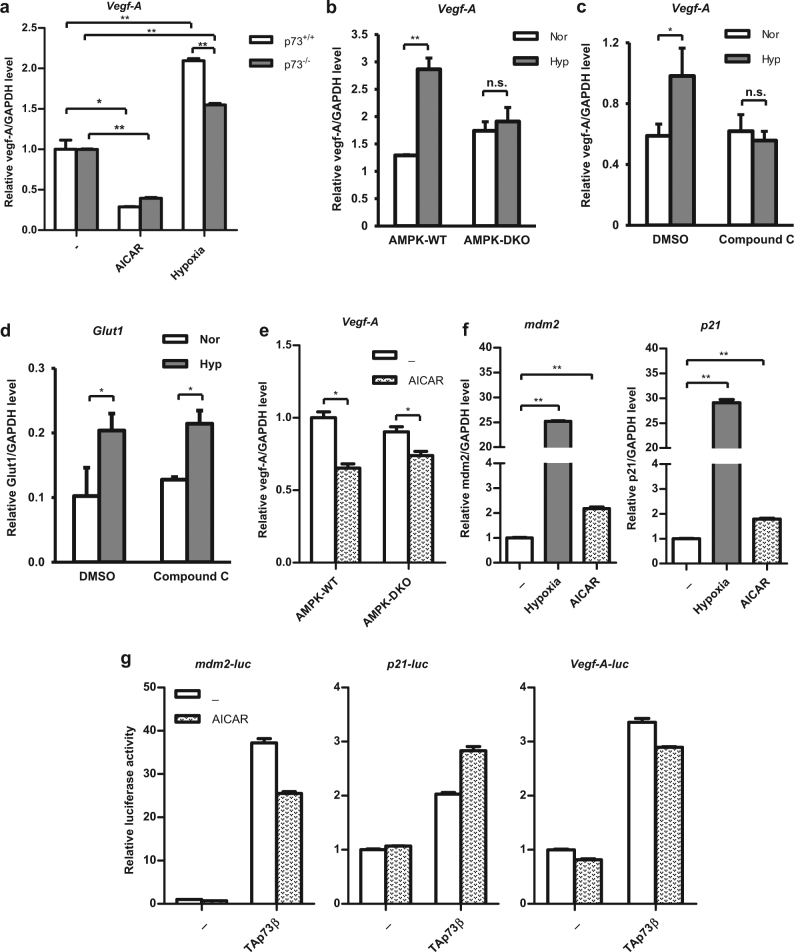
Vegf-A target gene transactivation by hypoxia requires AMPK, but AMPK activation alone is not sufficient for Vegf-A transactivation. **a**, **b** Expression of *vegf-A* was determined in WT or p73^−/−^ MEFs after exposure to hypoxia or treatment with 1 mM AICAR for 24 h by real-time qPCR (**a**), and similarly in AMPK-WT or AMPK-DKO MEFs (**b**). Error bars indicate S.D. *p < 0.05; **p < 0.01. n.s. not significant. **c**–**f** Levels of *vegf-A* (**c**) and *glut-1* (**d**) in H1299 cells upon hypoxia treatment in the presence of compound C were determined as described. Similarly, *vegf-A* expression was determined in AMPK-WT and AMPK-DKO MEFs cells upon 1 mM AICAR treatment (**e**). Moreover, *mdm2* and *p21* expression in H1299 cells was determined after exposure to hypoxia or AICAR for 24 h by real-time qPCR (**f**). Relative levels of target genes to *gapdh* are shown. Error bars indicate S.D. *p < 0.05; **p < 0.01. n.s. not significant. **g** Activation of *mdm2*, *p21*, and *vegf-A* promoter-luciferase activity was determined in H1299 cells transfected with TAp73β, and exposed to AICAR. All real-time PCR experiments were repeated two to three times. All luciferase experiments were repeated two to three times, and representative data from an experiment is shown

As AMPK activation by AICAR led to TAp73 stabilisation, albeit independent of the HIF-1α-Siah1 axis that also contributes to TAp73 stabilisation, we investigated the role of AMPK pathway on the transactivation of *vegf-A*, which is induced by TAp73 under hypoxia.^12,13^ While hypoxia resulted in the elevation of *vegf-A*, AICAR treatment did not, and even led to its suppression (Fig. 5a), though AICAR alone was found to be sufficient to induce TAp73 stabilisation (Fig. 2). However, inhibition of AMPK signalling, by using AMPK-DKO cells or treatment with compound C, resulted in abrogation of *vegf-A* activation by hypoxia in both cases (Fig. 5b, c), without having any significant effects on the expression of the other HIF-1α hypoxic target gene such *glut-1* (Fig. 5d), highlighting the requirement for AMPK in the hypoxic response that is pertinent for *vegf-A* activation. In addition, *vegf-A* activation was consistently reduced in both AMPK-WT and AMPK-DKO cells upon AICAR treatment (Fig. 5e). This data also allude to the possibility that AICAR-mediated direct AMPK activation alone is insufficient for potentiation of TAp73s’ transactivation activity to regulate *vegf-A* expression. Consistently, while *vegf-A* activation by hypoxia was compromised in TAp73 null MEFs as earlier shown^12^ (Fig. 5a), absence of TAp73 had no significant effects on *vegf-A* down-regulation upon AICAR treatment (Fig. 5a). To further confirm that AICAR treatment is insufficient to induce TAp73’s transactivation potential, we evaluated the activation of several target genes. Though hypoxia activated *p21* and *mdm2* expression significantly, AICAR treatment only led to a very modest activation of these genes (Fig. 5f), indicating that TAp73’s transactivation potential may not be sufficiently modulated by direct AMPK activation alone. Furthermore, AICAR treatment also did not potentiate TAp73-mediated activation of *p21*, *mdm2* and *vegf-Α* promoters (Fig. 5g), further confirming that AMPK-mediated TAp73 stabilisation is insufficient to potentiate the latter’s transactivation activity. Together, these results suggest a bifurcation of signalling pathways from TAp73 stabilisation to its angiogenic target gene activation.

### Tube formation is enhanced by hypoxia but not by AICAR treatment

**Fig. 6:**
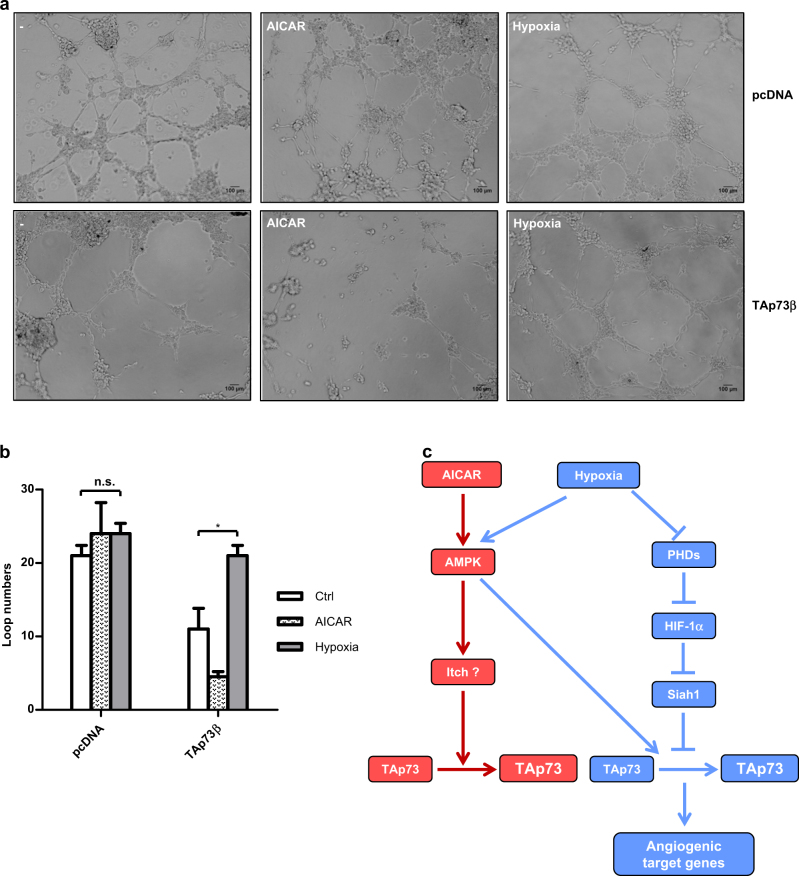
Hypoxia activated supernatant from TAp73-expressing cells promote endothelial tube formation and proposed model for the role of AMPK in hypoxia-mediated TAp73 stabilisation and activation. **a** HUVECs were grown in supernatants from H1299 cells expressing empty vector or TAp73β and treated with either AICAR or hypoxia. Representative pictures are shown at the point of harvest. The quantification of loop numbers is shown in **b**. Error bars indicate S.D. The tube formation experiments were repeated three times. *p < 0.05. n.s. not significant. **c** Under normal conditions, direct AMPK activation by AICAR results in the stabilisation of TAp73 (left, red arrows), which is independent of HIF-1α and repression of *Siah1*, and does not require S426 phosphorylation. Upon hypoxia, AMPK is activated concomitant to and independent of HIF-1α (right, blue arrows). While AMPK activation upon hypoxia contributes to TAp73 stabilisation and consequent TAp73-mediated *vegf-A* activation, its activation and upregulation of TAp73 alone (independent of hypoxia) by AICAR is insufficient to mediate TAp73-dependent transactivation of *vegf-A*. Together, the model proposes a scenario in which multiple signalling pathways induced by hypoxia results in the concerted stabilisation and activation of TAp73 function leading to angiogenic gene transactivation, represented by *vegf-A*. Stimulation of one pathway alone, i.e. AMPK, though capable of leading to TAp73 stability, is insufficient for its activity, thereby decoupling the processes of TAp73 stability and activation. ?: represents yet to be uncovered pathways

To evaluate the functional consequences of TAp73 stabilisation by hypoxia and through direct AMPK activation by AICAR, we performed in vitro tube formation assays using HUVEC endothelial cells, which are sensitive to angiogenic factors like *vegf-Α*.^31,32^ We thus evaluated if hypoxia-mediated, TAp73β-dependent *vegf-Α* production will have an impact on the ability of endothelial cells to form tubes. To this end, supernatants of H1299 cells transfected with vector or TAp73β, and exposed to hypoxia or treated with AICAR were used to stimulate HUVEC cells. Supernatant from vector transfected cells did not have major effects on the tube formation ability of HUVEC cells (Fig. 6a). However, supernatant from TAp73β-transfected cells that were exposed to hypoxia significantly stimulated tube formation, as determined by numbers of loops formed by the extending tubes. By contrast, supernatant from TAp73β-transfected cells treated with AICAR led to considerable reduction in tube formation and viability of the HUVEC cells (Fig. 6a, b). These data therefore correlate with the earlier observations that *vegf-Α* expression was induced by TAp73β only upon hypoxia, but not AICAR. Together, these data support the notion that direct AMPK activation, though capable of stabilising TAp73β, is insufficient to induce the latter’s angiogenic transactivation potential and thus, functional activity.

## Discussion

The data presented here demonstrates two salient points about AMPK’s role in TAp73 stabilisation. Firstly, AMPK activation, either *in silo* or in the context of hypoxia, leads to the stabilisation of TAp73, though the mechanisms appear to be different between the two scenarios. In the hypoxic context, the E3 ligase Siah1 appears to be the critical regulator of TAp73 (Fig. 6c). Nonetheless, abrogation of AMPK signalling attenuates TAp73 stabilisation upon hypoxia, suggesting that AMPK contributes to this process in other ways, which need to be uncovered. On this note, AMPK has been suggested to support HIF-1α mediated direct transactivation of the angiogenic genes,^37–39^ which may be operative in this instance independent of *Siah1*. On the contrary, AMPK activation alone – in the absence of hypoxia, appears to regulate TAp73 stabilisation independent of Siah1 repression by HIF-1α. Thus, these data highlight a scenario in which the context of AMPK activation determines the signalling pathways that regulate TAp73 stabilisation (Fig. 6c).

Secondly, AMPK activation by AICAR, though capable of inducing TAp73 stabilisation, appears not to be relevant for TAp73-mediated *vegf-A* transactivation, in contrast to the hypoxic context. Thus, TAp73 stabilisation alone may not be sufficient to induce its transcriptional activity, and thus requires other co-operative signals transduced by hypoxia signalling, alluding to a two-step model for activation of TAp73’s angiogenic properties. An earlier report had suggested that AICAR treatment results in the activation of classical p53 target genes, which could be inhibited by the expression of a dominant-negative (DN) p73 (ref. ^25^), indicating that TAp73 may have a role in this process. However, these experiments were performed in HCT116 colon tumour cells, which contain a WT p53. AICAR was also shown to be incapable of inducing these targets in p53 null HCT116 cells, similar to our findings in the p53 null H1299 cells (Fig. 5f). Moreover, overexpression of DN-p73 was found to reduce endogenous p53 levels in that study. Thus, AICAR-mediated regulation of p53 target gene expression appears likely to be due to the presence of p53 rather than due to TAp73.

These data are also consistent with the dual role of AMPK activation in regulating tumour cell proliferation. AMPK activation has been suggested to inhibit tumour cell growth, and this may likely reflect *in silo* activation of the AMPK signalling pathway, which then leads to p53-dependent cell growth arrest.^22–24^ On the contrary, AMPK activation in the contexts of cellular survival, in this case represented by hypoxia, contributes to p53-independent TAp73 activation and consequent angiogenic target gene transactivation. Thus, a collective model that emerges is that AMPK activation alone is not supportive of growth, and may lead to tumour suppression, be it through TAp73 stabilisation or p53 activation. By contrast, AMPK activation in contexts like hypoxia leads to the exhibition of TAp73’s pro-angiogenic properties, together supporting cellular growth. This is further supported by our data showing that supernatant from TAp73-expressing cells exposed to hypoxia is capable of inducing tube formation in HUVEC endothelial cells, but not those from similar cells treated with AICAR.^40^

An open question is how does AMPK activation lead to TAp73 stabilisation? Reports by Adamovich et al. indicated that AMPK phosphorylated TAp73 on Serine 426 to regulate its stability.^25^ Based on the data presented in this study using the TAp73 S426A mutant, as well as the truncation mutations lacking this residue, it is relatively clear that S426 is not required for its stability, either by AICAR treatment or upon hypoxia. However, the partial attenuation of TAp73 stabilisation by DN-AMPK under hypoxia suggests that AMPK activity contributes to this process. Whether AMPK directly phosphorylates TAp73 on other sites, or indirectly regulates TAp73 stabilisation requires further investigation. The former possibility is however unlikely as there are only two major AMPK phosphorylation sites predicted earlier, and the other site (S374) appears to be irrelevant as well.^25^ Moreover, whether the E3 ligase Itch has a direct role is questionable as we observed that TAp3 can indeed be stabilised by AICAR treatment in Itch^−/−^ cells. Thus, the mechanisms of AMPK-mediated TAp73 stabilisation requires further investigation.

Finally, this work highlights a paradigm for transcription factor activation. While stabilisation and activation of transcription factors occur upon cellular stimulation, these two processes are often thought to be inter-related and coupled.^41^ However, the data presented here suggests that TAp73 stabilisation by AMPK, through AICAR as an inducing agent, does not have the required wherewithal to transactivate *vegf-A*, indicating that the two processes can indeed be decoupled. While we do not yet know what is the second signal for activation of TAp73’s transactivation potential, hypoxic signalling is certainly capable of inducing that to facilitate TAp73’s ability to activate *vegf-A* expression. Further elucidation of the mechanistic details will enable us to potentially selectively target the growth promoting and angiogenic activity of TAp73, leaving it in a stable state, which could be exploited to enhance its apoptotic properties to induce cell death and decrease tumour growth.
