# Correction to: context-dependent AMPK activation distinctly regulates TAp73 stability and transcriptional activity

**DOI:** 10.1038/s41392-021-00504-8

**Published:** 2021-04-24

**Authors:** Dan Li, Iqbal Dulloo, Kanaga Sabapathy

**Affiliations:** 1 Division of Cellular & Molecular Research, Humphrey Oei Institute of Cancer Research, National Cancer Centre Singapore, Singapore, Singapore; 2 Cancer and Stem Cell Biology Program, Duke-NUS Graduate Medical School, Singapore, Singapore; 3 Department of Biochemistry, Yong Loo Lin School of Medicine, National University of Singapore, Singapore, Singapore; 4 Institute of Molecular & Cellular Biology, Singapore, Singapore; 5 Present Address: Sir William Dunn School of Pathology, University of Oxford, Oxford, UK

**Keywords:** Cancer therapy, Lung cancer

Correction to: *Signal Transduction and Targeted Therapy* (2018) **3**, 20; 10.1038/s41392-018-0020-y, published online 27 July 2018

The author of this published article^1^ reported that a funding source was omitted in the acknowledgement, and want to add below funding source.
